# Muscular visualisation on a bone scan in paraneoplastic dermatomyositis associated with breast cancer

**DOI:** 10.11604/pamj.2016.23.9.7455

**Published:** 2016-01-22

**Authors:** Abdelhamid Biyi, Abderrahim Doudouh

**Affiliations:** 1 Department of Nuclear Medicine, Mohammed V Military Teaching Hospital, Mohammed V University of Rabat, Morocco

**Keywords:** Dermatomyositis, bone scan, paraneoplastic syndrome, muscular uptake

## Image in medicine

Bone scintigraphy is widely used for initial staging and subsequent follow-up of oncologic patients. 99mTc-labelled diphosphonates also have the ability to detect nonosseous disorders. Muscular uptake on bone scintigraphy has been reported in various conditions including rhabdomyolysis, inflammatory muscle disease, traumatic myositis, polymyositis and dermatomyositis. The proposed mechanism of radiotracer uptake is binding to mitochondrial calcium which is increased in the damaged muscle cells. Here, we report a case of a 62 years old woman with a past history of mastectomy for a galactophoric breast carcinoma, without additional adjuvant treatment, who complained of generalized muscle pain and weakness, erythematous rash on the face, the neck and upper arms. Creatine kinase level was elevated (462 IU/L; normal <200 IU/L). Antinuclear antibodies were negative. These findings with the electromyographic examination (myopathic changes) supported the diagnosis of dermatomyositis. A bone scan performed as a part of the staging process showed diffuse and intense muscular uptake of 99mTc-MDP in the shoulder girdles, both proximal upper and lower limbs corresponding to the sites of symptomatic muscles. No evidence of skeletal involvement was detected (A). PET-CT images showed hypermetabolic foci in multiple thoracic lymph nodes (C) and the left iliac bone (D). A punch biopsy confirmed the diagnosis of metastatic lymph node from a breast carcinoma. Immunosuppressive therapy based on oral prednisone and endoxan followed by molecular targeted therapy (Herceptin and taxotere) improved the myositis and cutaneous eruption. Tree months later, creatine kinase level and muscular uptake of 99mTc-MDP dramatically decreased (B).

**Figure 1:**
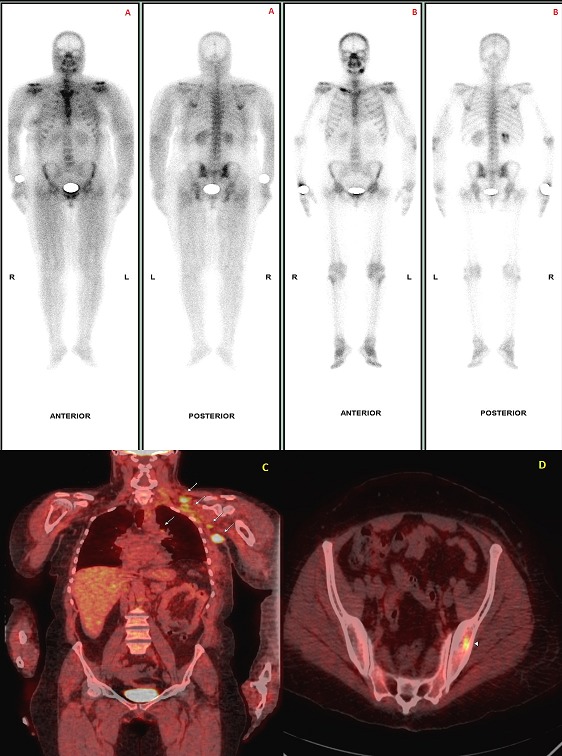
A) bone scan showing diffuse and intense muscular uptake of 99mTc-MDP in the shoulder girdles, both proximal upper and lower limbs corresponding to the sites of symptomatic muscles. Neither upper limb bones nor the lowers are demonstrated. B) bone scan performed one year after. The patient has no muscular or dermatologic manifestation. Scintigraphic abnormalities are no longer detected (the right clavicle increased uptake is related to a traumatic origin). C) F18-FDG PET-CT coronal fusion image: pathological lymph nodes are identified as FDG-avid lesions in the left axilla, supraclavicle fossa and mediastinum (white arrows). Histology confirmed the nodal metastases. D) F18-FDG PET-CT fusion image: Axial views showing a hypermetabolic focus in the left iliac bone (arrowhead) subsequent with skeletal metastases

